# In Vitro Characterization of Biodegradable Polyurethane Foams With Facile Gelatin Modification for Traumatic Wound Hemostasis and Regeneration

**DOI:** 10.1002/jbm.a.37982

**Published:** 2025-10

**Authors:** Natalie Marie Petryk, Mary Beth B. Monroe

**Affiliations:** Biomedical and Chemical Engineering and BioInspired Syracuse: Institute for Material and Living Systems, Syracuse University, Syracuse, New York, USA

**Keywords:** degradable biomaterials, gelatin, hemostatic dressing, polyurethane, tissue scaffold, wound healing

## Abstract

Polyurethane (PUr) foams are widely explored for embolic, hemostatic, and tissue engineering applications. Their tunable pore structure, mechanical properties, and degradation rates make PUr foams ideal scaffolds for thrombus formation and cell infiltration. Despite their embolic and hemostatic efficacy, PUrs are entirely synthetic, which limits their long-term healing capacity to facilitate tissue regeneration. To improve PUr-driven healing, this work explores the facile modification of biodegradable PUr foams with bioactive gelatin through simple physical and chemical incorporation methods accomplished post-foam fabrication. The gelatin-modified PUr foams had increased platelet interactions and quicker clotting times than the unmodified PUr foams due to the procoagulant nature of gelatin. Furthermore, the gelatin-modified foams had significantly improved cell attachment, spreading, and proliferation of fibroblasts on foam pores, which could translate to enhanced wound repair through tissue migration into the PUr scaffold. Overall, the simple modification of biodegradable PUr foams with bioactive gelatin can significantly improve healing outcomes in traumatic wounds and various regenerative tissue applications.

## 1 | Introduction

Polyurethane (PUr) foams are widely explored for embolic and hemostatic applications because they are biocompatible, hemocompatible, durable, and easily scalable [1, 2]. Their high porosity and low density allow them to be crimped into a low-profile shape for easy delivery into small-diameter vasculature or deep tissues. For example, PUr foams have been employed for the embolization of aneurysms, tumors, and abnormal connections like arteriovenous malformations (AVMs) and fistulas [3–8]. Their high porosity allows crimping to small devices that can be delivered through a catheter, followed by expansion in situ to block the target vessel. Then, the expanded scaffold can occlude blood flow and provide the structural framework for thrombus formation [9–11]. Embolic PUr foams can be biostable for permanent embolization or biodegradable for temporary occlusion. Additionally, PUr foams have demonstrated efficacy as a hemostatic dressing to control bleeding in traumatic wounds [12, 13]. They can induce rapid, localized clotting in deep, irregularly shaped, non-compressible wounds. A biodegradable PUr foam dressing could eliminate wound rebleeding associated with dressing removal and further serve as scaffolding to promote healing and tissue regeneration.

Beyond traumatic wound regeneration, biodegradable PUr foams have vast potential to serve as tissue engineering scaffolds for a range of tissue types. PUrs are highly tunable biomaterials, making it easy to tailor the architecture, mechanical properties, and degradation rates, all of which heavily affect cell infiltration, vascularization, and tissue integration [14–18]. Interconnected, open-cell foams make it easier for cells and tissue to migrate into the polymer network and allow for the exchange of nutrients and waste [19, 20]. The mechanical strength of the material can affect cell interactions and how well the material withstands stress and pressure from blood flow [21, 22]. Controlled biodegradation eliminates the need for surgical removal to reduce patient risks and healthcare costs. Biodegradable PUr scaffolds can safely degrade over time into biocompatible byproducts without long-term foreign body reactions [23]. Their degradation rate can be tailored to match the rate of tissue formation and ensure structural integrity until new tissue is formed [24]. PUr foams can also mimic the natural extracellular matrix (ECM) by providing a temporary framework that supports cell attachment, migration, proliferation, and differentiation [25]. As the foams degrade, cells can replace the scaffold with their own ECM.

Traditional PUr foams are relatively biostable because urethane bonds are hydrolytically stable. Many PUrs include oxidatively labile tertiary amines or ethers whose oxidation rate is relatively slow in implantable applications, allowing for longer-term polymer biostability [18, 26]. Hydrolytically labile esters have been incorporated into the backbone of PUr foams to increase foam degradation rates, but these were limited by lengthy synthesis and purification methods or slow degradation [17, 27–29]. Recent work incorporating both oxidatively labile sulfides and hydrolytically labile esters into the network through a simple click-chemistry reaction demonstrated a significant increase in foam degradation rates, offering easy-to-synthesize scaffolds that degrade within clinically relevant time frames for tissue repair in traumatic wounds [30].

Additionally, bioactive PUr foams have been explored to improve healing and increase cell interactions on PUr scaffolds within the native tissue environment. In previous work, bioactive gelatin and collagen were physically or chemically incorporated into biostable PUr foam scaffolds because of their procoagulant and cell-adhesive properties [31]. The bioactive components increased foam-induced clotting, which can help decrease the time to achieve hemostasis in traumatic wound healing. The bioactive PUr foams also increased cell attachment, spreading, and proliferation on PUr foam pores, which could help to promote tissue repair.

This work explores the physical and chemical incorporation of bioactive gelatin into biodegradable PUr foams to enhance blood–and cell-material interactions for future application in traumatic wound hemostasis and healing. Gelatin is a procoagulant that can decrease the time to achieve thrombus and hemostasis. With controlled biodegradation, the foams could then provide scaffolding for cell infiltration, during which gelatin could increase cell attachment and proliferation for effective tissue remodeling. Ultimately, biodegradable PUr foams with bioactivity could provide effective temporary scaffolds for improving embolic and hemostatic treatments with potential for future use in a range of tissue engineering applications.

## 2 | Methods

### 2.1 | Materials

N,N,N′,N′-tetrakis-(2-hydroxy-propyl)-ethylenediamine (HPED), triethanolamine (TEtA), hexamethylene diisocyanate (HDI), dibutyltin dilaurate (DBTDL), triethylamine (TEA), hydrogen peroxide (H_2_O_2_, certified ACS, 30%), and ethanol (reagent alcohol) were purchased from Fisher Scientific (Waltham, MA) and used as received. Trimethylolpropane tris(3-mercaptopropionate) (TMPMP), 2-hydroxyethyl acrylate (HEA), 1,4-diazabicyclo[2.2.2]octane (DABCO 33 LV), lithium phenyl-2,4,6-trimethylbenzoylphosphinate (LAP) photoinitiator, glutaraldehyde (25%), and formaldehyde (≥ 36%) were purchased from Sigma Aldrich (St. Louis, MO) and used as received. Vorasurf DC 198 surfactant was provided by DOW (Midland, MI). Phosphate-buffered saline (PBS), Dulbecco’s phosphate-buffered saline (DPBS), gelatin (type A, 175 bloom), methacrylic anhydride, Dulbecco’s modified Eagle’s medium (DMEM), penicillin–streptomycin (P/S), and fetal bovine serum (FBS) were purchased from Thermo Fisher Scientific (Waltham, MA) and used as received. Na-citrated whole porcine blood was purchased from Lampire Biological Laboratories (Pipersville, PA).

### 2.2 | Initial Screening of Degradable Foams Based on Clotting Analysis

The “biostable” control PUr containing slow-oxidizing tertiary amines ([12, 14]) and the three fastest degrading foam formulations previously studied (30T-1,2, 30T-2,2, and 30H-2,2, [30]) containing fast-oxidizing sulfides and hydrolytically labile esters were first analyzed to understand their clotting behavior under dynamic blood flow. A direct perfusion model previously employed and discussed later in Section 2.14.5.1 was used to pump Na-citrated whole porcine blood through the foams (*n* = 3, 8 mm diameter, 2.5 cm length) at a rate of 40 mL/min for 10 min [31]. The clotting behavior of the foams was determined based on the amount of blood that passed through the samples versus the amount of blood that was rerouted due to clotting (Figure S1). The 30T-2,2 foam formulation had the best clotting behavior, based on having redirected the most blood flow. For this reason, the 30T-2,2 foam was selected as the “biodegradable” foam for the remaining studies.

### 2.3 | Synthesis of Degradable Polythiol

The degradable polythiol used during the synthesis of the 30T-2,2 foam was made as previously described [30]. Briefly, 1 mol of TMPMP was mixed with 3 mols of HEA and 1 wt% TEA at 3500 rpm for 30 s. Attenuated total reflectance Fourier transform infrared (ATR-FTIR) and ^1^H nuclear magnetic resonance (NMR) spectroscopy were used to confirm the final product [30].

### 2.4 | Foam Synthesis

The “biostable” control PUr and the “biodegradable” 30T-2,2 PUr foams were made as previously described [14, 17, 30]. Briefly, an isocyanate (NCO) premix (1 NCO mol equivalent [HDI] and 0.35 hydroxyl (OH) mol equivalents [HPED and TEA or the degradable polythiol]) was prepared inside a controlled atmosphere glovebox and placed in a 50°C oven to react for 48 h. After 48 h, Vorasurf DC 198 surfactant was added to the NCO premix. An OH mix was prepared with the remaining 0.65 OH mol equivalents, deionized (DI) water, and catalysts (DBTDL and DABCO 33 LV). The OH mix was combined with the NCO mix, speed-mixed at 1800 rpm for 5 s, quickly poured into a 400 mL cylindrical mold, and transferred to a 50°C oven to foam for 5 min. The added weight percent of each foaming component is summarized in Table 1. After polymerization, foams were washed in two changes of DI water and 70% ethanol to remove catalysts and surfactant. Then, they were dried under vacuum before all characterization and analysis.

### 2.5 | Physical Incorporation of Gelatin

Gelatin was dissolved in DPBS at 200 μg/mL by heating the solution to 50°C under constant stirring at 300 rpm. PUr foams were sterilized in 70% ethanol for 1 h and then incubated in DI water overnight to fully remove the ethanol. In a sterile biosafety cabinet, the samples were added to the gelatin solution to incubate for 1 h at 37°C. Then, the gelatin-coated samples were removed from the solution and rinsed with sterile PBS [31].

### 2.6 | Chemical Incorporation of Methacrylated Gelatin

Methacrylated gelatin (gelMA) was prepared as previously reported [31, 32]. To incorporate gelMA into the PUr foams, gelMA was dissolved in DPBS at 0.08 mg/mL at 60°C. After dissolution, 0.2 wt% of LAP photoinitiator was added and quickly dissolved using a vortexer. Sterile foam samples (~20 mg) were placed in a 24-well plate and incubated in 1 mL of the gelMA solution for 5 min. After 5 min, the foams were removed from the solution and gently pressed to remove excess gelMA. The samples were placed in a fresh 24-well plate and cured in a UV light box (365 nm) for 3 min.

### 2.7 | Spectroscopic Analysis

To confirm the incorporation of gelatin and gelMA into the biostable and biodegradable foams, the surface chemistry of foam cross sections was characterized using a Nicolet i70 ATR-FTIR spectrometer (Fischer Scientific, Waltham, MA). Small foam pieces (~2 mg) were scanned at a resolution of 4 cm^−1^. Spectra were generated using OMNIC software and presented as absorbance vs. wavelength over an average of 16 scans.

### 2.8 | Foam Pore Structure

Foam samples (~1 cm^3^, *n* = 3) were coated in Au at 45 mA for 45 s using a high vacuum sputter coater (Denton Vacuum, Moorestown, NJ). A JEOL JSM-IT100 scanning electron microscope (SEM, JEOL USA, Peabody, MA) was used to image pore morphology and the incorporated bioactive components at 10 kV and 50× and 300× magnification. Pore size and pore interconnectivity were quantified using ImageJ and GNU Image Manipulation Program (GIMP), respectively [14].

### 2.9 | Density

Cylindrical foam samples (*n* = 3, ~2.5 cm length, 8 mm diameter) were weighed. The volume was calculated from sample length and average diameter (taken from 3 measurements across foam length). Density was calculated from the mass and volume.

### 2.10 | Thermal Analysis

The glass transition temperature (*T*_g_) of foam samples (*n* = 3, 3–5 mg) was measured in their dry and wet (plasticized) states using a DSC 250 differential scanning calorimeter (DSC, TA instruments, New Castle, DE) following heating/cooling cycles previously reported [14, 18]. The *T*_g_ was determined from the half-height transition of the final heating cycle.

### 2.11 | Mechanical Testing

The compressive modulus was measured for dry and wet (plasticized) cylindrical samples (*n* = 3, 8 mm diameter, 4 mm thickness). Foam samples were plasticized in DI water at 50°C for 10 min. For each test, the strain rate was controlled at −1 mm/min, and the end limit was set to 24 N. The load (N), sample area (mm^2^), position (mm), and sample length (mm) measurements outputted from the test program were used to calculate stress (kPa) and strain (mm). Compressive modulus was determined from the slope of the linear region of the stress vs. strain curve between 0.02 and 0.1 strain.

### 2.12 | Swelling Ratio

The dry mass ($W_{d}$) of cylindrical foam samples (*n* = 3, ~1 cm length, 8 mm diameter) was measured. The samples were incubated in DI water for 24 h and then gently patted to remove excess water. The swollen mass ($W_{s}$) of each sample was measured, and the swelling ratio (SR) was calculated as:

$$SR=\frac{W_{s}-W_{d}}{W_{d}}$$

### 2.13 | In Vitro Degradation

Samples (8 mm diameter, 2.5 cm length, *n* = 3) were prepared for a degradation study in 3% H_2_O_2_. The initial mass of each sample was recorded before adding each foam to individual scintillation vials with 10 mL of media. The samples were incubated at 37°C with shaking at 100 rpm. Every 3 days, the samples were removed from the media, dried overnight in a room-temperature vacuum oven, and weighed to determine mass loss. The samples were imaged to record erosion profiles and then returned to fresh media to continue incubating/shaking for a total of 45 days.

### 2.14 | Blood-Material Interactions

#### 2.14.1 | Hemolysis

Hemolysis was tested on foam samples (*n* = 3, 10 mg) with Na-citrated whole porcine blood as an initial measure of hemocompatibility to ensure that the foams would not rupture red blood cells (RBCs) [30]. DI water and 1× PBS were used as negative (hemolytic) and positive (non-hemolytic) controls, respectively. The optical absorbance of sample supernatant was measured with a plate reader (BioTek Synergy 2, Agilent Technologies, Winooski, VT) at 545 nm. Percent hemolysis was calculated as:

$$\text{Hemolysis}\;(\%)=\frac{{OD}_{\text{sample}\;}-\;{OD}_{\text{negative}\;\text{control}\;}}{{OD}_{\text{positive}\;\text{control}\;}-\;{OD}_{\text{negative}\;\text{control}\;}}*100$$

#### 2.14.2 | Blood Absorption

Dry foam samples (*n* = 3, ~1 cm^3^) were weighed ($W_{d}$), placed in a 24-well plate, and incubated in 2 mL Na-citrated whole porcine blood at 37°C. After 1 h, samples were weighed to determine their swollen weight ($W_{d}$), and blood absorption was calculated as:

$$\text{Absorption}\;(\%)=\frac{W_{b}-W_{d}}{W_{d}}*100$$

#### 2.14.3 | Platelet Attachment

A lactase dehydrogenase (LDH) cytotoxicity assay kit (Cayman Chemical, Ann Arbor, MI) was used to quantify platelet attachment to the PUr foams (*n* = 3, ~20 mg) [17, 30]. Briefly, the foams were incubated in 1 mL Na-citrated whole blood at 37°C for 30 min, gently rinsed with PBS to remove nonattached platelets, and soaked in 10% Triton X-100 for 1 h to lyse the attached platelets. Then, 100 μL of sample supernatant was transferred to a 96-well plate, and 100 μL of the LDH reaction solution was added to each well. After incubating for 30 min at 37°C, a microplate reader (BioTek Synergy 2, Agilent Technologies, Winooski, VT) was used to measure the absorbance values at 490 nm. Platelet concentrations for each sample were determined based on a standard curve from absorbance values of known platelet concentrations [30].

#### 2.14.4 | Clotting Time

A coagulation assay was performed as previously reported [14, 17, 18, 30, 31]. Recalcified blood was added to foam samples (*n* = 4, ~5 mg) at room temperature for 0, 6, 12, and 18 min. After each time point, 1 mL of DI water was added to each sample for 5 min to lyse any free RBCs, then centrifuged at 10,000 rpm for 5 min. Lysate (200 μL) was collected from each tube and added to a 96-well plate. The amount of hemoglobin released from the lysed RBCs was measured at an absorbance of 540 nm using a microplate reader (BioTek Synergy 2, Agilent Technologies, Winooski, VT).

#### 2.14.5 | Clotting Under Dynamic Blood Flow

##### 2.14.5.1 | Direct Perfusion In Vitro Flow Model.

A direct perfusion model previously described was employed to study the effects of the bioactive components on foam-induced clotting under dynamic blood flow in vitro [31]. Briefly, the model has a peristaltic pump to control blood flow rate, a flow chamber to test the foam sample, pressure gauges on either side of the flow chamber, and an “outpour” and “overflow” container to collect blood that passes through the sample and blood that is rerouted around the sample, respectively [31]. To begin perfusion, 500 mL of Na-citrated whole porcine blood was warmed to 37°C using a water bath. Foams were perfused with the warmed blood at 40 mL/min for 10 min. After 10 min, the fluid volume in each container was measured, and the sacrificial flow chamber tubing was cut proximal and distal to the foam sample. The samples were carefully removed from the tubing and weighed to determine their swollen weight. Then, the samples were gently rinsed with PBS to remove non-adherent cells. Each sample was cut into 6 equal cylindrical cross-sections along the length of the foam: 3 cross sections—1 from the proximal, middle, and distal end of the foam relative to the direction of flow—were fixed in 2.5% glutaraldehyde for platelet imaging using SEM, and the other 3 were fixed in 4% formalin for histological analysis. Bleach was perfused through the system to remove blood before subsequent test runs, followed by PBS for priming.

##### 2.14.5.2 | Platelet Imaging.

After 24 h in 2.5% glutaraldehyde, the foam cross-sections were dehydrated in increasing ethanol concentrations (50%, 70%, 95%, and 100%) for 30 min each, then dried in a vacuum oven overnight. The dried samples were sputter-coated with Au at 45 mA for 60 s and imaged using SEM (1000× magnification, 10 kV).

##### 2.14.5.3 | Histological Analysis.

After 24 h in 4% formalin, the foam cross-sections were processed and prepped on glass slides as previously reported [31]. The dried slides were deparaffinized, rehydrated, and stained with hematoxylin and eosin (H&E) and phosphotungstic acid hematoxylin (PTAH). A Leica DM300 microscope was used to image slides using a 4× and 40× objective. Fibrin was quantified for each field of view at 4× from an average of *n* = 6 sections per cross-section (proximal, middle, and distal) by isolating the PTAH-stained fibrin using the color-select tool and histogram analysis feature in GIMP. Fibrin percentage was calculated from the total pixel area of fibrin relative to the total pixel area of the sample in each field of view.

### 2.15 | Cell Interactions

#### 2.15.1 | Cytocompatibility

Cell viability was determined using NIH/3T3 Swiss mouse fibroblasts (ATCC-CCL92). The cells were cultured as previously described [30, 31]. Cells were seeded in a 24-well plate at 10,000 cells/mL (600 μL/well) and incubated for 24 h. Then, sterile foam samples (*n* = 3, 6 mm diameter, 2 mm thickness) were placed into Transwell inserts (6.5 mm, 0.4 μm pore polyester membrane, Corning) and added to the cell-seeded wells. After 24, 48, and 72 h of sample incubation, the inserts, samples, and cell media were removed from each well. Then, 600 μL of 10% alamar blue (resazurin) was added to the sample-containing wells and control wells (*n* = 3, positive: cells; negative: no cells). After incubating at 37°C for 2 h, 150 μL from each well was transferred to a 96-well solid black polystyrene plate. Fluorescence (excitation: 530/25; emission: 590/35; position: top 50%) was measured using a microplate reader (BioTek Synergy 2, Agilent Technologies, Winooski, VT), and cell viability was calculated as:

$$\text{cell}\;\text{viability}\;(\%)=\frac{\text{Average}\;\text{flourescence}\;\text{of}\;\text{test}\;\text{group}\;}{\text{Average}\;\text{flourescence}\;\text{of}\;\text{positive}\;\text{control}\;}*100$$

#### 2.15.2 | Cell Attachment

Cell attachment to PUr foams was determined at 24, 48, and 72 h. NIH/3T3 cells with green fluorescent protein (GFP) were cultured and resuspended in reduced-serum media (DMEM, 2% FBS, 1% P/S) to control for cell attachment to foams due to serum protein adsorption. After counting cell concentration with a hemocytometer, the required volume (X) of cell suspension to achieve a final concentration of 50,000 cells/well was determined for a total cell suspension of 200 μL/well [31]. This volume X was droplet seeded directly onto sterile foam samples (*n* = 3 for each time point, 6 mm diameter, 2 mm thickness) in a 96-well plate and set to incubate at 37°C. After 1 h, reduced-serum media was added at a volume of 200 – *X* μL to each well to reach a final concentration of 50,000 cells/well and returned to the incubator. A Leica Thunder microscope was used to view cell attachment after 24, 48, and 72 h. Z-stack images were acquired at 10× magnification, and ImageJ was used to measure the total cell count.

#### 2.15.3 | Cell Spreading

Foam samples (*n* = 3 for each time point, 6 mm diameter, 2 mm thickness) were droplet-seeded as described above (Section 2.15.2) with NIH/3T3 cells without GFP. At 24, 48, and 72 h, samples were removed from culture media, prepped, and stained with phalloidin and DAPI [31]. The samples were rinsed with PBS and imaged using an inverted microscope at 20× magnification. After fluorescent imaging, the samples were prepared for SEM by fixing in 2.5% glutaraldehyde for 24 h at 4°C. The samples were dehydrated in ethanol (50%, 70%, 95%, and 100%; 30 min each) and dried in a vacuum oven overnight. SEM micrograph images were taken to view cell morphology (10 kV and 200× magnification). Adobe Photoshop was used to colorize the cells in each image. GIMP was used to quantify cell spreading using the color-select tool (as described in Section 2.14.5.3) by determining the pixel area coverage of cells.

### 2.16 | Statistical Analysis

Measurements are presented as mean ± standard deviations. A Student’s *t*-test was performed in Microsoft Excel with two-tailed distribution and two-sample unequal variance. If *p* < 0.05, differences were considered statistically significant. All foam formulations were compared to the “standard” unmodified biostable foam, previously reported as the “control” PUr (*p < 0.05) [17, 18, 30, 33]. Additionally, the modified biodegradable foams (gelatin and gelMA) were compared against the unmodified biodegradable foam to understand the effects of the bioactive components on material properties and biological interactions (^#^*p* < 0.05).

## 3 | Results

### 3.1 | Incorporation of Bioactive Components

**FIGURE 1 |:**
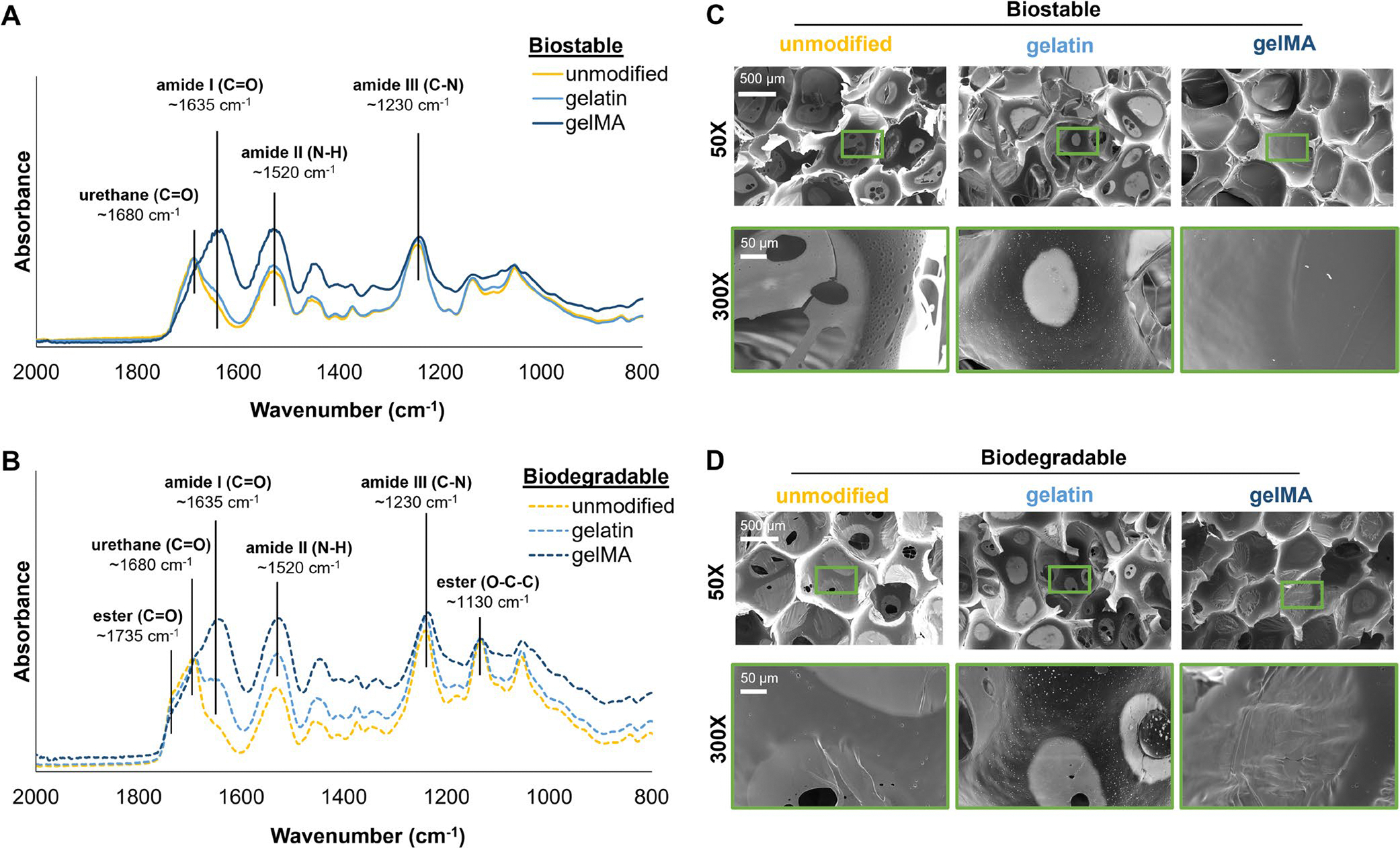
FTIR spectra of the (A) biostable and (B) biodegradable foams confirming the physical and chemical incorporation of gelatin. SEM micrographs of the (C) biostable and (D) biodegradable foams at 5× and 30× magnification showing the overall pore structure, physical adsorption of gelatin, and gelMA incorporation. Scale bars apply to all images of the same magnification.

The successful incorporation of gelatin and gelMA into the PUr foams was confirmed in the FTIR spectra and SEM micrographs that show pore morphology (Figure 1). The biostable and biodegradable foams each have a peak from the C═O bond of urethanes around 1680 cm^−1^, while the biodegradable foams have additional peaks at 1735 and 1130 cm^−1^ corresponding to the C═O and O—C—C bonds of esters, respectively (Figure 1A,B). The incorporation of gelatin into the PUr foams is best determined by the amide I (C═O) peak; the physically incorporated gelatin foam samples have a shoulder off of the urethane peak corresponding with amide I at 1635 cm^−1^, while gelMA-containing foams have a much stronger, fuller peak (Figure 1A,B) [34, 35]. The shoulder peak is larger in the biodegradable samples than in the biostable samples; the esters and sulfides in the biodegradable foams make the foams more hydrophobic [30], which may preferentially allow more gelatin to adsorb to the surfaces [36, 37]. Amide II and amide III groups have peaks that overlap with the N—H and C—N of urethanes at 1520 and 1230 cm^−1^, respectively (Figure 1A,B) [34, 35, 38].

Both the unmodified biostable and biodegradable foams have relatively closed-cell pores (~800 μm diameter, ~3% pore interconnectivity), with some pore membrane thinning (lighter contrast areas) and pore interconnectivity (pinhole openings) as seen in Figure 1C,D and quantified in Table 2. The physical adsorption of gelatin on the biostable and biodegradable foam pore surfaces can be visualized as bead-like structures in the SEM images (Figure 1C,D). The chemical incorporation of gelMA into the biostable and biodegradable foams is confirmed by smoother, closed-cell pores due to the crosslinked gelMA network filling foam pores (Figure 1C,D), resulting in essentially 0% pore interconnectivity (Table 2). The gelMA foam samples also have a smaller average pore size, which could be explained by gelMA shrinkage after drying (Table 2).

### 3.2 | Physical, Thermal, and Mechanical Analysis

**FIGURE 2 |:**
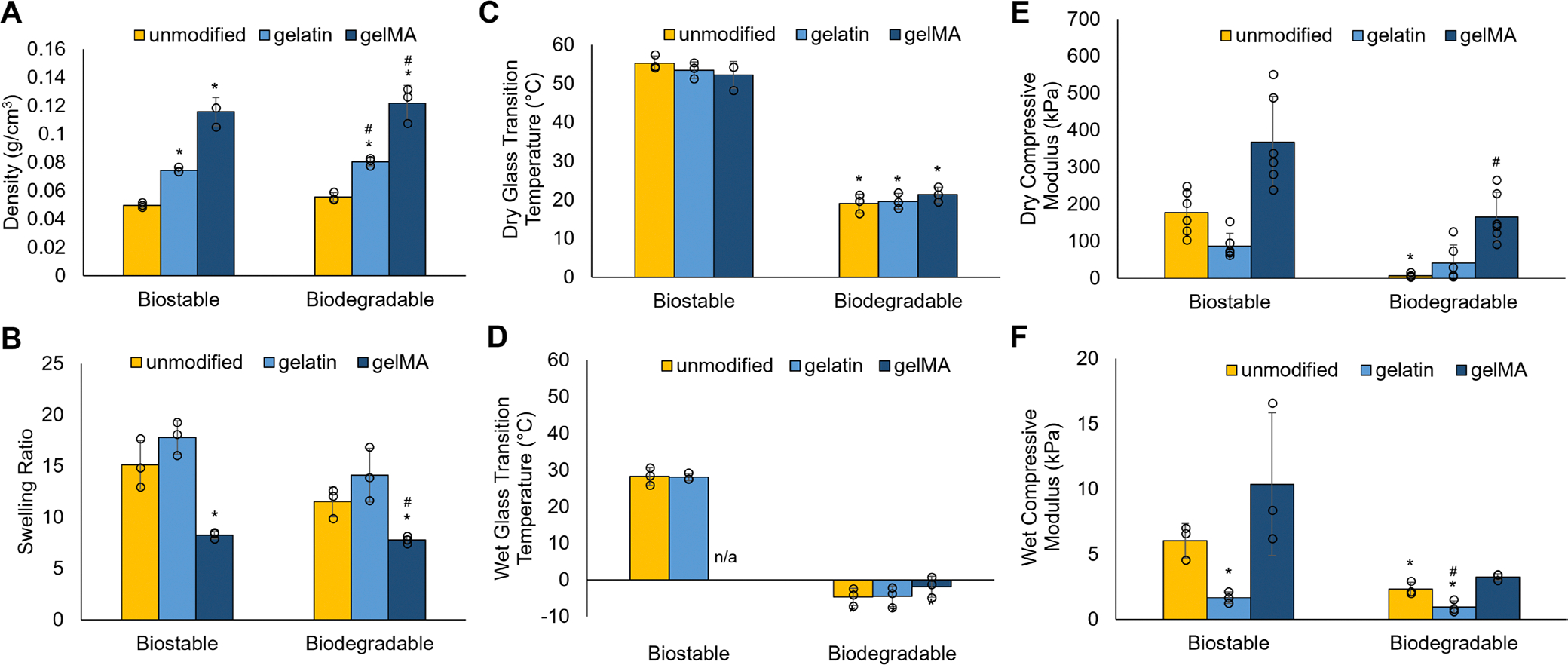
Foam physical, thermal, and mechanical properties. (A) Density of foams in their open-porous shape. (B) Swelling ratio after 24-h in DI water (37°C). Glass transition temperature of (C) dry and (D) wet (plasticized) samples. Compressive modulus of (E) dry and (F) wet (plasticized) samples. *p < 0.05 compared to unmodified biostable foam; ^#^*p* < 0.05 compared to unmodified biodegradable foam (A–D, F: *n* = 3; E: *n* = 6).

The unmodified biostable and biodegradable foams have comparable foam densities, consistent with their similar pore sizes (Figure 2A). The gelatin and gelMA modified samples have significantly higher densities than the unmodified foams, with the gelMA samples having the highest density due to the additional crosslinked network throughout the samples. The unmodified and gelatin foams have densities < 0.1 g/cm^3^, indicating low-density foams, while the gelMA samples are > 0.1 g/cm^3^. This increased density could make it more difficult to crimp gelMA samples into a low-profile shape for implantable applications. For both the biostable and biodegradable samples, the gelatin foams have slightly increased swelling ratios compared to the unmodified foams. In contrast, the gelMA foams have significantly lower swelling, consistent with previous work (Figure 2B) [31]. The biodegradable foams have significantly lower glass transition temperatures in dry and wet conditions than the biostable foams due to longer backbone chains that reduce the required energy to rotate bonds (Figure 2C,D) [30]. However, the gelatin and gelMA modifications did not significantly affect thermal properties (Figure 2C,D). The biostable gelMA sample’s *T*_g_ cannot be measured in its wet, plasticized state because of a large melting peak consuming the *T*_g_ (Figure S2). The biostable foam is relatively more hydrophilic than the biodegradable foam, which could make it more difficult to fully remove excess water retained in the gelMA before measuring *T*_g_, resulting in the melting peak. The unmodified biodegradable foam has a significantly lower compressive modulus than the unmodified biostable foam in its dry state (Figure 2E), consistent with previous work [30]. Incorporating gelMA into the biodegradable scaffold significantly increases its modulus, making it comparable to the biostable foam. The compressive modulus of the wet samples is more relevant to the forces that would be experienced in vivo. In its plasticized state, the unmodified degradable foam has a significantly lower modulus than the unmodified biostable foam, and the gelatin modification further decreases the compressive modulus (Figure 2F).

### 3.3 | In Vitro Degradation

**FIGURE 3 |:**
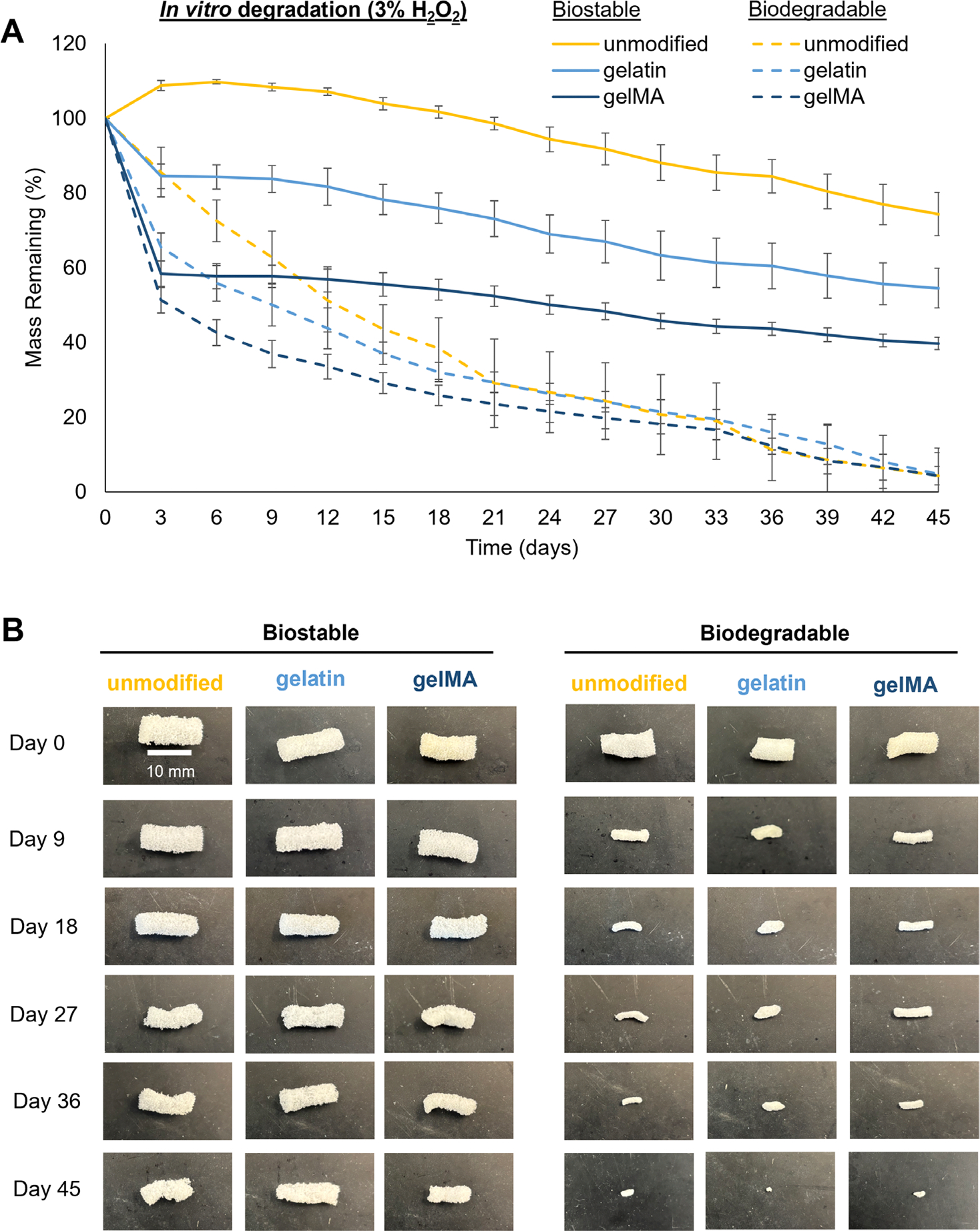
In vitro degradation in 3% H_2_O_2_. (A) Foam sample mass loss over 45 days (*n* = 3). (B) Erosion profiles of foams throughout degradation. Scale bar applies to all images.

To understand the effects of the bioactive modifications on foam degradation, mass loss was measured for foam samples over a 45-day degradation study in 3% H_2_O_2_ (Figure 3). A 3% H_2_O_2_ solution mimics “real-time” in vivo degradation rates, providing both oxidative and hydrolytic degradation of the foams in vitro [30]. Among the biostable foams, the unmodified foam had ~74% mass remaining at 45 days, compared to ~55% and ~40% among the gelatin and gelMA foam samples, respectively (Figure 3A). The gelatin and gelMA samples had the largest decrease in mass within the first 3 days. Then, their degradation slowed down and experienced comparable rates of linear mass loss: unmodified (0.37 ± 0.04 mg/day; *R*^2^ = 0.9819), gelatin (0.44 ± 0.02 mg/day; *R*^2^ = 0.9852), and gelMA (0.39 ± 0.04 mg/day; *R*^2^ = 0.9789). Gelatin is hydrophilic, so both its physical and chemical incorporation into the foams could make it easier for water to initially access polymer chains in each sample and cause foam breakdown. Alternatively, the gelatin/gelMA may be degrading/solubilizing within the first 3 days. More in-depth degradation studies would be required to understand what is happening. That said, cells have previously attached, spread, and proliferated on foam pores over a 3-day period [31], so even if the bioactive modification is not stable beyond 3 days in vivo, it should still be long enough to facilitate and support cell attachment, during which cells can begin laying down their own ECM.

Similar trends were noted among the biodegradable foams, where there was initially a greater decrease in mass among the gelatin and gelMA foam samples (Figure 3A). The degradation rates were faster than that of the biostable foams, but they still experienced linear mass loss after 3 days: unmodified (0.72 ± 0.06 mg/day; *R*^2^ = 0.9303), gelatin (0.64 ± 0.03 mg/day; *R*^2^ = 0.9580), and gelMA (0.68 ± 0.06 mg/day; *R*^2^ = 0.9604). All the biodegradable foams had comparable mass remaining at 45 days at roughly 4%–5%. The erosion profiles of representative foam samples can be seen in Figure 3B. Both the biostable and biodegradable foams underwent surface erosion, as demonstrated by the maintained structural integrity of each foam. The biostable foams did not change noticeably over 45 days, while the biodegradable foams had a significant size reduction that corresponded with their near 100% degradation (Figure 3B).

### 3.4 | Static Blood-Material Interactions

**FIGURE 4 |:**
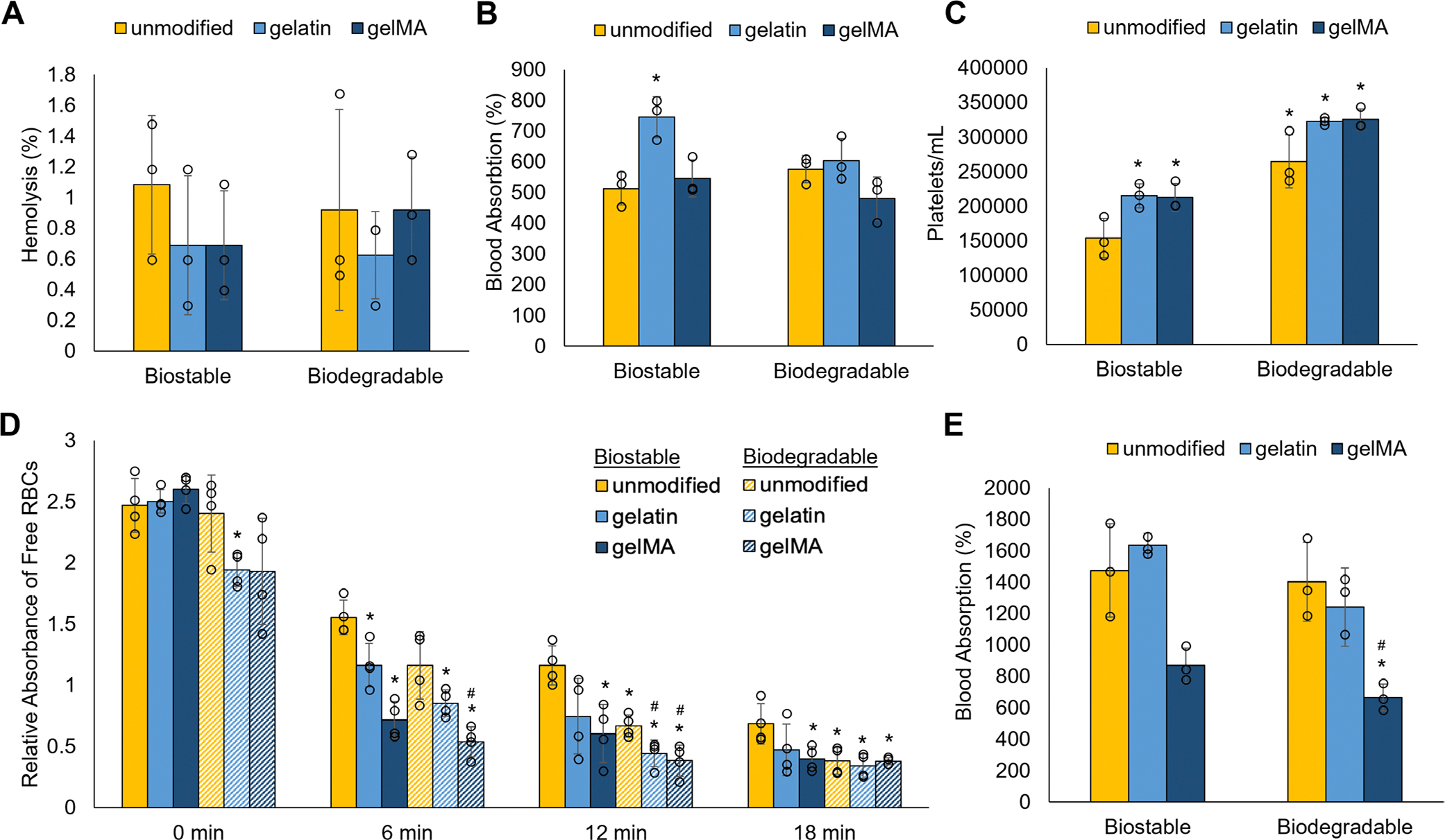
Static blood-material interactions and blood absorption after perfusion. (A) Hemolysis of each foam after 1 h incubation in Na-citrated whole porcine blood. (B) Weight percent of blood absorption after 1 h incubation in Na-citrated whole porcine blood. (C) Concentration of attached platelets to foams measured using the LDH assay. (D) Foam coagulation times based on free red blood cells (RBCs). (E) Weight percent of blood absorbed during 10-min perfusion runs. *p < 0.05 compared to the unmodified biostable foam; ^#^*p* < 0.05 compared to the unmodified biodegradable foam (A–C, E: *n* = 3; D: *n* = 4 compared within each time point).

As a first measure of hemocompatibility, the foams were tested for whether they were hemolytic and would rupture RBCs. All the foams are non-hemolytic with less than 1.5% hemolysis (Figure 4A). All the foams have comparable blood absorption, except for the gelatin biostable foam, which absorbed significantly more blood (Figure 4B). The gelatin and gelMA biostable foams had significantly more platelet attachment than the unmodified biostable foam, consistent with their procoagulant effect (Figure 4C). The unmodified biodegradable foam also had greater platelet attachment than the unmodified biostable foam as measured by the LDH assay, consistent with previous findings due to the foam’s greater hydrophobicity [30]. The greater number of platelets attached to these foams is reflected in faster and more effective clotting in the coagulation assay (Figure 4D). By 6 min, the gelatin and gelMA biostable foams had significantly fewer free RBCs, indicative of increased clotting, compared to the unmodified biostable foam. By 12 min, the unmodified biodegradable foam also had significantly reduced free RBCs, while the gelatin and gelMA biodegradable foams had even fewer. The relative absorbance of free RBCs among the bioactive foams and the unmodified biodegradable foam plateaued between 12 and 18 min, indicating that a stable clot had formed.

### 3.5 | Dynamic Blood-Material Interactions

**FIGURE 5 |:**
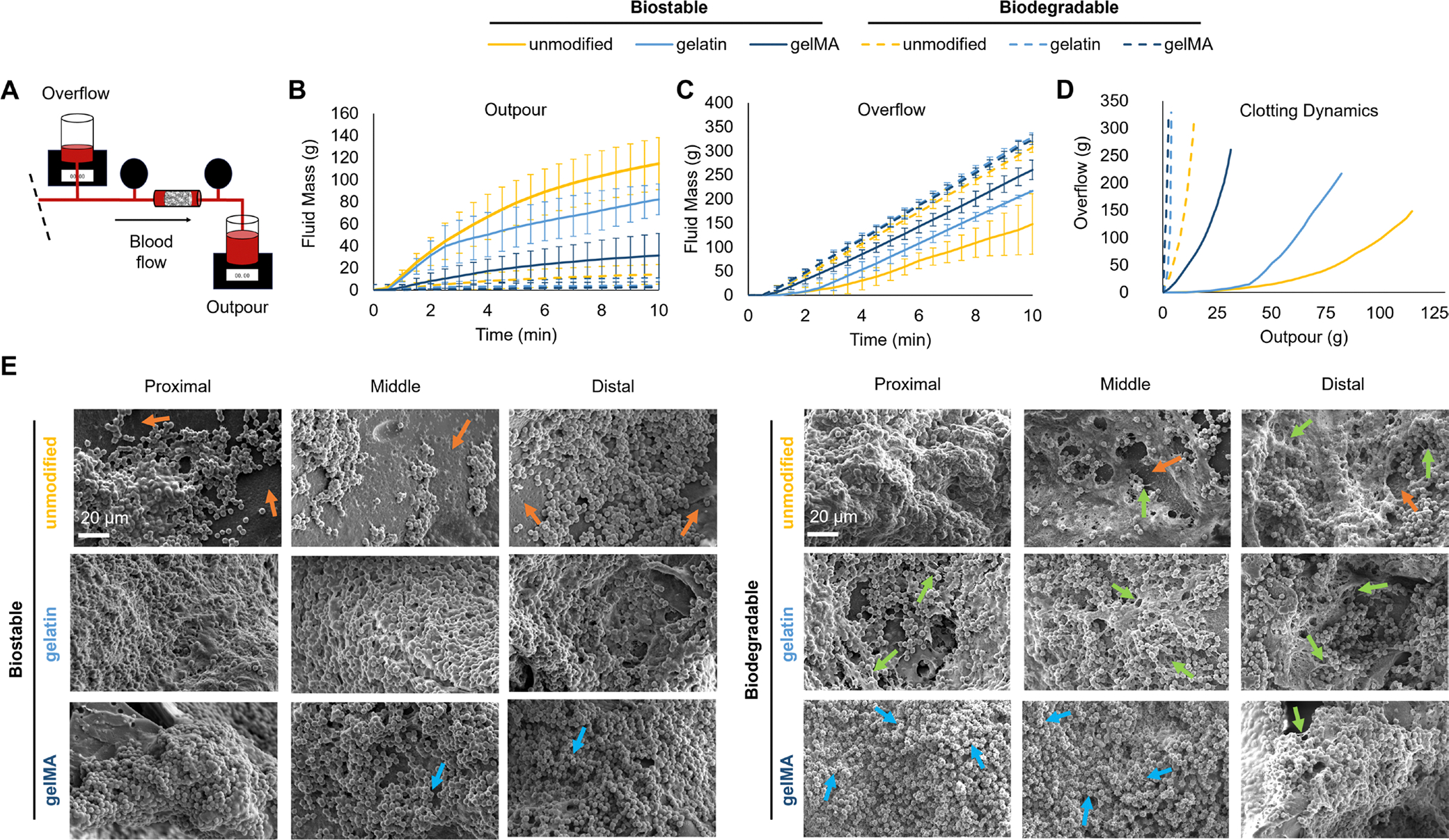
Dynamic clotting behavior of foams after perfusion with Na-citrated whole porcine blood for 10 min. (A) Schematic representation of the outpour and overflow containers in the perfusion setup. (B) Fluid mass passed through each sample collected in the outpour container. (C) Fluid mass rerouted into the overflow container. (D) Overall clotting behavior of each foam based on rerouted blood versus blood through each sample. (E) SEM images of platelet interactions on foams at proximal, middle, and distal locations relative to the direction of blood flow (blue arrows: activated platelets; green arrows: fibrin; orange arrows: foam surface). Scale bar applies to all images.

While the static blood-material interactions offer valuable insight into the clotting behavior of each foam, dynamic blood-material interactions provide a better understanding of how these foam-based dressings may initiate clotting under hemorrhaging conditions in vivo [31]. The amount of blood absorbed by each sample during the perfusion runs (Figure 4E) follows similar trends to the amount of absorbed blood under static conditions (Figure 4B). By comparing the amount of blood that passes through the sample (outpour) and the amount of blood rerouted (overflow), we can determine the clotting behavior of each foam (Figure 5A). The procoagulant nature of the gelatin increases the clotting accomplished by the gelatin and gelMA biostable foams compared to the unmodified biostable foam, as seen by less fluid passing through the samples, that is, collected in the outpour container (Figure 5B), and more fluid rerouted around the samples, that is, collected in the overflow container (Figure 5C). The unmodified biodegradable foam has significantly increased clotting compared to the unmodified biostable foam, so while gelatin and gelMA increased the clotting of the biodegradable foams, it is to a lesser extent (Figure 5B,C). Figure S3 shows the blood flow rates into each container, providing insight into clotting time. For example, the unmodified and gelatin biostable foam had the largest increase in flow rate into the overflow container in the first 2 min, after which the rate slowed down/plateaued, suggesting that a stable clot began to form around 2 min. This trend is seen around 1.5 min in the gelMA biostable and unmodified biodegradable foams, and at roughly 1 min for the gelatin and gelMA biodegradable foams, indicating quicker times to stop bleeding (Figure S3). The overall clotting dynamics are shown in Figure 5D, showing improved clotting among the bioactive foams compared to their corollary unmodified foams, with the greatest amount of clotting among the gelatin and gelMA biodegradable foam. Despite the increase in clotting and amount of redirected blood flow, the pressure differential across each sample remained similar (Figure S3).

The perfused samples were then analyzed for platelet interactions and fibrin deposition. In response to vascular injury, blood platelets will adhere to the damaged tissue, activate, and recruit more platelets to the injury site, forming aggregated “platelet plugs” [39, 40]. This platelet plug is stabilized by a polymerized fibrin mesh, initiated by a series of clotting factors, to create a temporary seal to stop bleeding [40, 41]. Platelet morphology is a key indicator of this process. Non-activated platelets have a smooth, round surface, while activated platelets exhibit star-like protrusions known as filopodia.

The unmodified biostable foam has a slightly hydrophobic surface that promotes the attachment, activation, and aggregation of platelets [17, 18]. The unmodified biodegradable foam is more hydrophobic, which increases platelet interactions on the foam. Compared to the biostable foam, the biodegradable foam has larger platelet aggregates, as demonstrated by greater surface coverage in Figure 5E (orange arrows indicate the foam surface). Furthermore, there is evidence of fibrin deposition among the biodegradable foam (green arrows). The physical and chemical incorporation of gelatin into the foams further enhances their platelet interactions due to its procoagulant nature. There is greater platelet coverage and more aggregates formed on the gelatin and gelMA biostable foam samples. The most fibrin can be seen on the gelatin biodegradable foams (green arrows). The gelMA biostable foams have some platelets that are activated with small filopodia (blue arrows), while the gelMA biodegradable foams have significant activation among a majority of the platelets with more defined filopodia, particularly at the proximal and middle ends of the foam (Figure 5E).

**FIGURE 6 |:**
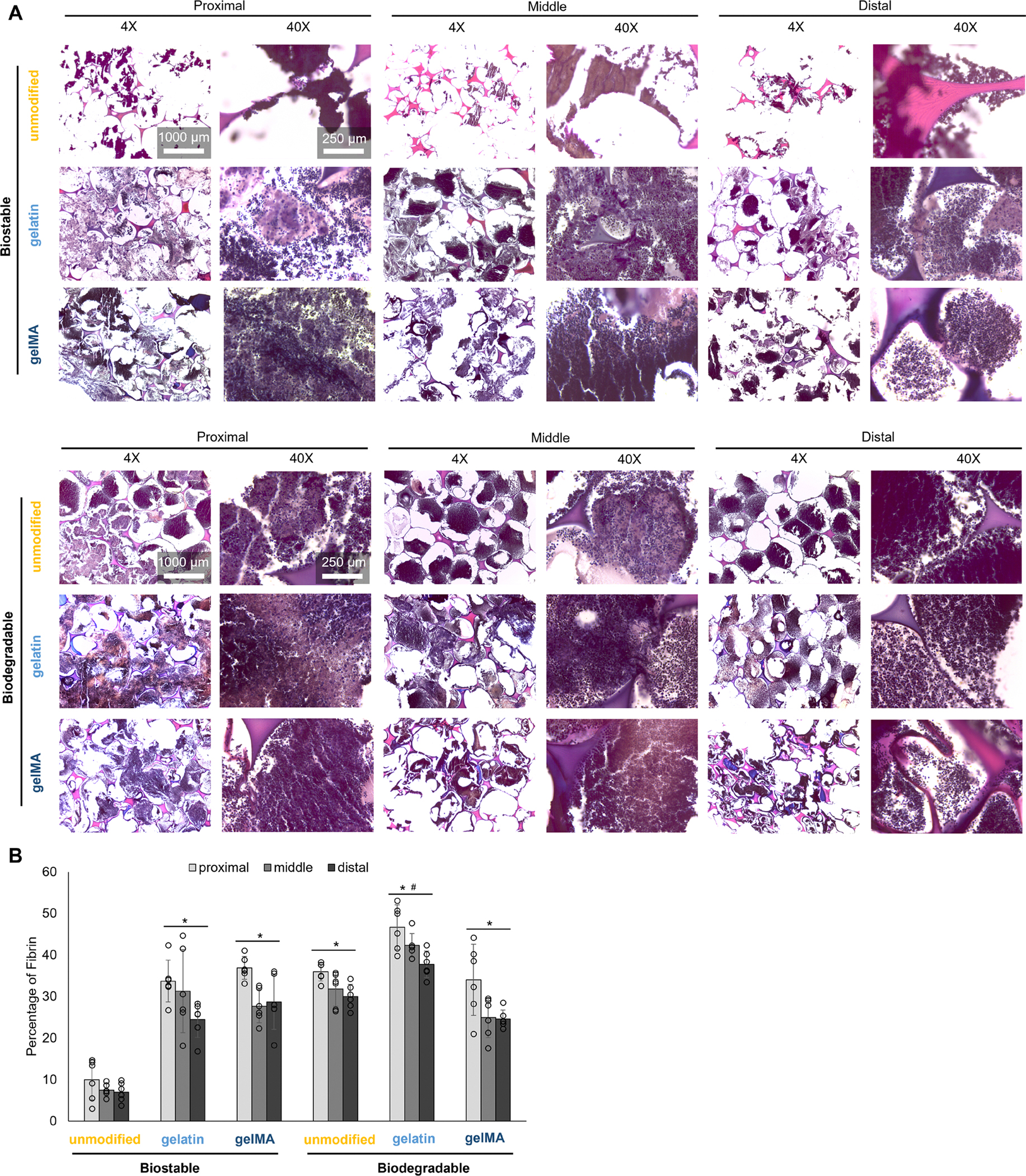
Blood-material interactions after 10 min of perfusion with Na-citrated whole porcine blood. (A) Fibrin deposition on PUr foams at the proximal, middle, and distal end relative to the direction of blood flow. Scale bar applies to all images of the same magnification. (B) Fibrin quantification based on the PTAH-stained samples. *p < 0.05 compared to the unmodified biostable foam; ^#^*p* < 0.05 compared to the unmodified biodegradable foam (*n* = 3).

The clotting behavior of each foam can further be explained by fibrin deposition on each sample. Histological staining distinguishes foam pore struts (pink from eosin) from endothelial cells (light purple from hematoxylin), while fibrin is stained a dark purple by the PTAH. The fibrin morphology appears as bead-like structures in higher magnification images (Figure 6A). The unmodified biostable foam has significantly lower fibrin deposition than the other foams throughout their bulk (Figure 6A,B). Among all other foams, fibrin is present throughout the sample volume, with the most fibrin present at the proximal end of each foam where blood would first contact the sample and initiate clotting. Interestingly, the unmodified biodegradable foam had comparable fibrin deposition to that of the bioactive biostable foams (Figure 6B), demonstrating effective clotting without requiring additional procoagulant agents. That said, the gelatin biodegradable foam had significantly more fibrin deposition than the unmodified biodegradable foam, showing that gelatin further improves clotting.

### 3.6 | Cell-Material Interactions

**FIGURE 7 |:**
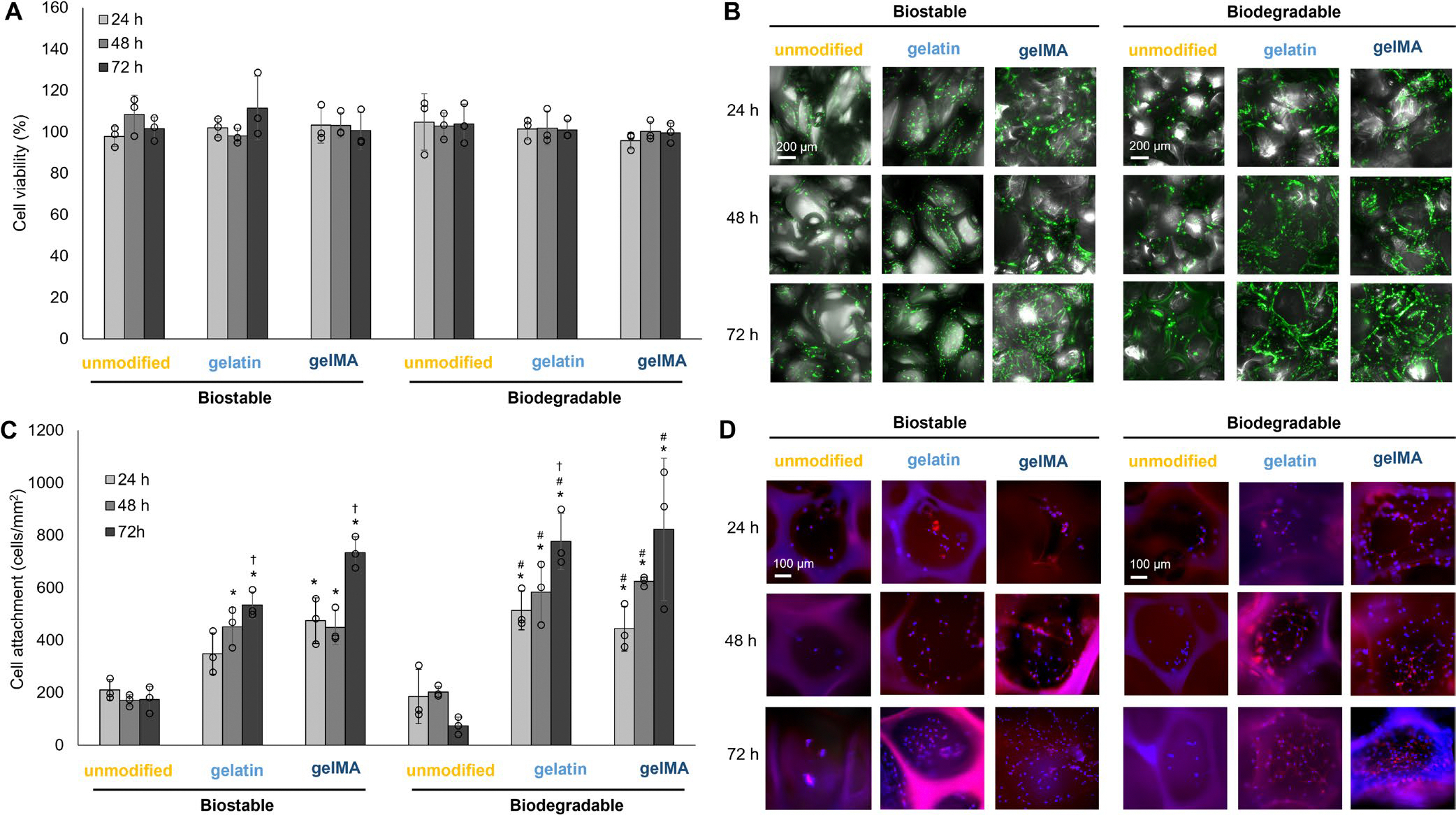
NIH/3T3 cell interactions on PUr foams. (A) Foam cytocompatibility over 24, 48, and 72 h relative to media-only cytocompatible control (*n* = 3). No statistically significant differences. (B) Merged z-stack brightfield and GFP images of cells attached to PUr foams. Scale bar applies to all images. (C) Cell attachment to foams over 24, 48, and 72 h. *p < 0.05 compared to the unmodified biostable foam; ^#^*p* < 0.05 compared to the unmodified biodegradable foam; ^†^*p* < 0.05 compared to the 24 h time point within a sample (*n* = 3). (D) NIH/3T3 cells on foams stained with DAPI (nuclei, blue) and phalloidin (actin, red) after 24, 48, and 72 h. Scale bar applies to all images.

All foams are highly cytocompatible with > 90% cell viability over 24, 48, and 72 h (Figure 7A), meeting the ISO 10993 biocompatibility standard for biomedical devices [42]. The z-stack merged brightfield and GFP images in Figure 7B demonstrate that cells are attached throughout the bulk of the foams. Some cells attach to the unmodified biostable and biodegradable foams initially, but these cells do not proliferate over the 72 h study (Figure 7C). Alternatively, the gelatin and gelMA foams (both biostable and biodegradable) have a greater number of cells initially attach at the 24 h time point, and these cells proliferate over 72 h (Figure 7B,C).

**FIGURE 8 |:**
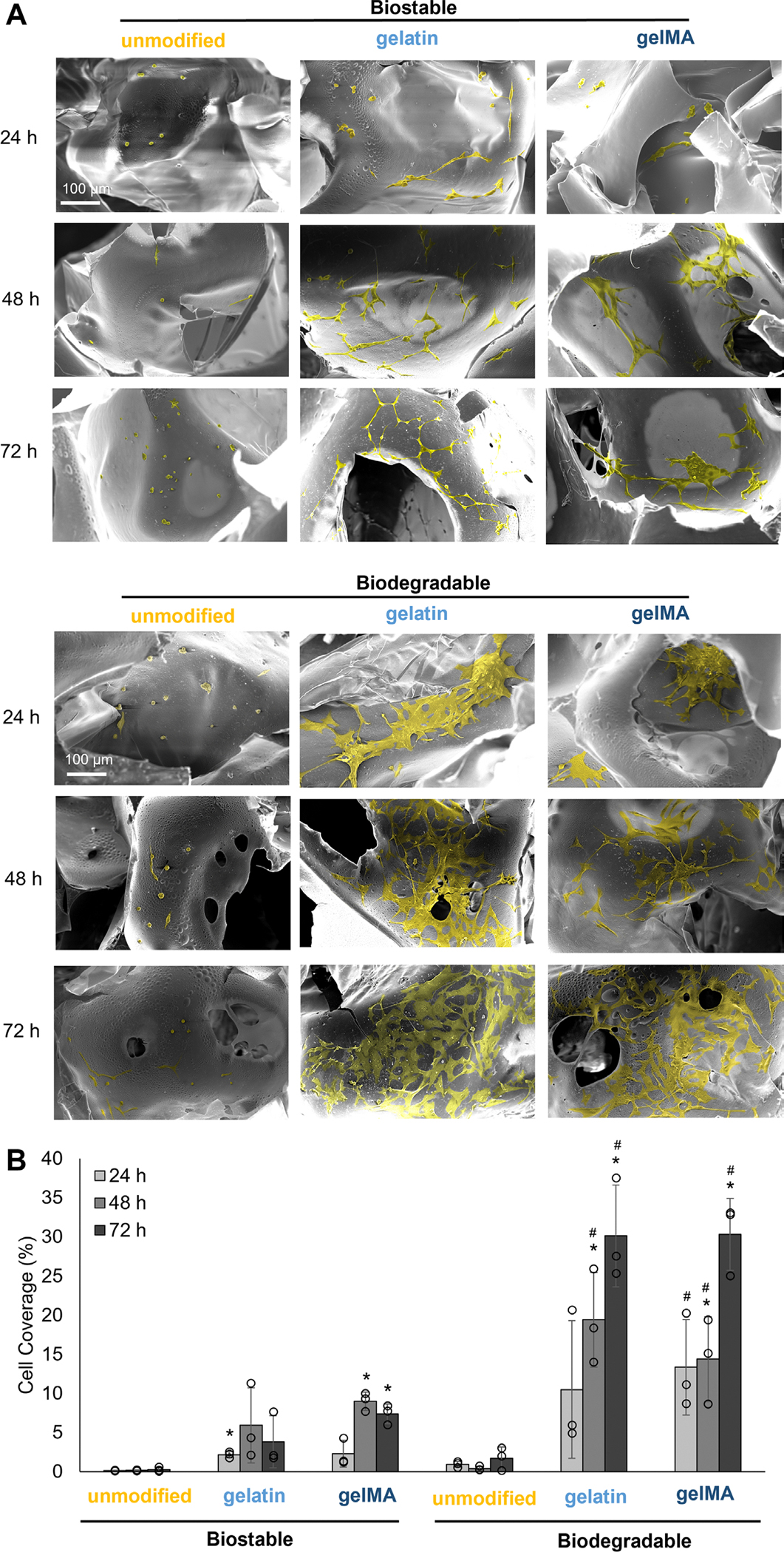
NIH/3T3 cell spreading on foam pores over 24, 48, and 72 h. (A) SEM images showing cell spreading (pseudo-colored yellow) on foam pores. Scale bar applies to all images. (B) Percent cell coverage on foam samples. *p < 0.05 compared to unmodified biostable foam; ^#^*p* < 0.05 compared to unmodified biodegradable foam (*n* = 3).

Figure 7D shows the merged images of the nuclei (stained blue by DAPI) and actin (stained red by phalloidin). The separate DAPI and phalloidin channels are included in Figure S4. These fluorescent images support the results seen among the samples with GFP cells, where there are fewer cells attached to the unmodified foams by 72 h, while the gelatin and gelMA foams show an increase in cell attachment. Qualitatively, the bioactive foams appear to have an increase in cell spreading over time (an increase in the red-stained actin). To confirm this finding, quantitative analysis was performed on SEM images to eliminate potential inaccuracies from foam autofluorescence (Figure 8).

While the DAPI and phalloidin stains confirm the nuclei and actin structure of each fibroblast, the SEM micrographs provide more detail into how the cells interact with the foams. Consistent with the GFP and DAPI/phalloidin images, the SEM images show that few cells attached to the unmodified biostable and biodegradable foams over 24, 48, and 72 h (Figure 8A). The cells are primarily balled up, with little cytoplasmic spreading. The gelatin and gelMA foams have significantly higher cell spreading and coverage on foam pores (Figure 8A,B). On the bioactive biostable foams, the fibroblasts are spindle-shaped with some cell-to-cell interactions (Figure 8A). On the bioactive biodegradable foams, the fibroblasts are more flattened and have greater cell-to-cell interactions. The significant increase in cell spreading and cell interactions on the bioactive biodegradable foams correlates with the increase in gelatin absorption observed in the FTIR spectra (Figure 1A,B).

## 4 | Discussion

This work has high potential to improve wound healing treatments for uncontrolled bleeding and tissue remodeling in traumatic wounds. It could be further expanded in future work to address broader embolic applications. While tissue repair and, therefore, cell interactions may not be as relevant to vessel occlusion, the gelatin-modified PUr foams in this work could improve current embolic treatments by reducing the time to achieve thrombus compared to entirely synthetic PUr foams, which rely solely on foam physical properties to induce thrombus [9–11].

Wound healing is a complex process involving four stages: hemostasis, inflammation, proliferation, and remodeling [43]. Hemostasis occurs immediately after injury to stop bleeding through platelet aggregation and clot formation via intrinsic, extrinsic, and common coagulation pathways [44–46]. Inflammation follows and can last up to a week, where neutrophils and macrophages clear pathogens and release cytokines to promote tissue repair [47–49]. The proliferation stage, occurring within 1–3 weeks, involves re-epithelization, angiogenesis, and granulation to rebuild ECM and tissue [39, 41, 50]. Remodeling, which can take months to years, strengthens the wound through collagen crosslinking and tissue maturation [47, 50]. In severe hemorrhaging and traumatic wound injuries, the body requires additional hemostatic agents or devices to stabilize bleeding and ensure proper wound healing.

Tissue engineering scaffolds can further facilitate wound repair and tissue regeneration.

Gelatin-based hemostatic dressings control bleeding effectively, but they are limited by their poor mechanical properties and poor control over degradation [51, 52]. PUr-based hemostatic dressings can improve upon these limitations because of their tunable pore structure, mechanical properties, and degradation rates [14, 16, 30]. They are highly effective at controlling bleeding, and they are inexpensive and easy to sterilize, manufacture, and scale up for commercialization [53, 54]. Despite the breadth of research into both PUr dressings and gelatin dressings for bleeding control, research into PUr-gelatin hybrid dressings for hemostatic applications is limited. Guo et al. developed a library of electrospun gelatin-modified PUr sponges that demonstrated successful hemostasis in a rat liver trauma model; however, these PUrs also contained chemically incorporated hemostatic agents, including tranexamic acid, which suppresses fibrin degradation and promotes clotting, and adenosine diphosphate, which induces platelet aggregation to promote clotting [55].

There is ample research into PUr-gelatin electrospun scaffolds for tissue engineering applications: Han et al. developed a PUr electrospun mat with gelatin and keratin that releases hydrogen sulfide to promote cell proliferation and angiogenesis [56]; Letha et al. synthesized an electrospun skin scaffold made of PUr and gelatin layers to mimic the epidermal and dermal layers, respectively [57]; and Abolhassani et al. designed an electrospun PUr-gelatin dressing loaded with honey and zinc oxide to increase antibacterial activity in a wound [58]. While all these electrospun PUr-gelatin scaffolds are designed for tissue remodeling, their use as a hemostatic agent was not explored. Additionally, electrospinning has many limitations that can hinder its commercial application, including low production yield and high production costs, inconsistent fiber diameters and fiber alignment, poor mechanical strength, and poor cell infiltration [59–61]. The gelatin-modified PUr dressings discussed in this work offer a novel wound-healing treatment option for achieving hemostasis and promoting cell interactions for potential tissue remodeling. The degradable polythiols are made using a rapid, solvent-free synthesis method, allowing for their immediate incorporation into PUr foams. The PUr foams are then easily modified with gelatin post-foam fabrication. The ease of both foam synthesis and surface modification can significantly improve scalability for clinical translation of a highly effective and affordable wound dressing.

This work demonstrated that the combined effect of a PUr and gelatin dressing can further improve wound healing outcomes. Both the biostable and biodegradable PUr foams explored in this work have previously demonstrated excellent hemostatic capabilities, with significant improvements compared to the commercially available QuikClot Combat Gauze (QCCG) [12, 30]. The simple modification of the PUr foams with gelatin and gelMA further enhanced foam-induced clotting, resulting in increased platelet attachment (Figure 4C), faster clotting times (Figure 4D), increased clotting behavior (Figure 5B–D), greater platelet activation (Figure 5E), and increased fibrin deposition (Figure 6A,B) Additionally, these bioactive biodegradable PUr foams demonstrated greater in vitro clotting performance compared to similar hemostatic PUr systems [14, 17, 18, 31]. Furthermore, the gelatin and gelMA PUr foams exhibited significantly increased cell attachment, spreading, and proliferation of fibroblasts on PUr foam pores (Figures 7 and 8), which can enable their use as tissue engineering scaffolds that facilitate the proliferation and remodeling stages of wound healing. Although only two different PUr formulations were explored in this work, the facile modification strategy can be applied to a range of potential foam formulations post-fabrication, eliminating the need to change established foam chemistries or foaming processes.

## 5 | Conclusions

This work offers a simple method for improving the bioactivity of biodegradable PUr foams for various tissue scaffolding applications, including traumatic wound hemostasis and healing. The physical and chemical incorporation of gelatin into the PUr foams significantly improved foam blood- and cell-material interactions in vitro. The increased platelet attachment, activation, and aggregation, and increased fibrin deposition demonstrated among the gelatin-modified PUr foams can ultimately decrease the time to achieve thrombus for embolic treatment and hemostasis in traumatic wounds. Similarly, the increase in cell attachment, spreading, proliferation, and cell-to-cell interactions among the bioactive scaffolds can enhance their capabilities in tissue engineering for wound remodeling. Future in vivo studies will be necessary to confirm these in vitro findings. Ultimately, the facile modification of these PUr foams with gelatin can provide effective, affordable dressings that are easy to scale up to improve embolic and hemostatic treatments and overall wound healing outcomes.

## Supplementary Material

Supporting Information

Additional supporting information can be found online in the Supporting Information section. **Data S1:** jbma37982-sup-0001-Supinfo.docx.

## Figures and Tables

**TABLE 1 |:** Synthesized PUr foam sample formulations provided as wt%.

Sample ID	HDI	HPED	TEtA	Degradable polythiol	Water	DBTDL	DABCO

Biostable	54.01	27.61	8.05	—	2.37	0.23	0.37
Biodegradable	39.84	20.37	—	29.61	1.75	0.46	0.64

**TABLE 2 |:** Pore structure analysis.

		Pore size (μm)	Interconnectivity (%)

Biostable	Unmodified	830 ± 100	3.0 ± 0.9
	Gelatin	820 ± 150	2.5 ± 0.9
	GelMA	720 ± 90*	0.0 ± 0.0*
Biodegradable	Unmodifed	820 ± 50	3.0 ± 0.1
	Gelatin	840 ± 90	3.1 ± 0.2
	GelMA	670 ± 80*^,#^	0.0 ± 0.0*^,#^

Note:

* *p* < 0.05 compared to unmodified biostable foam

# *p* < 0.05 compared to unmodified biodegradable foam (*n* = 3).

## Data Availability

The data that support the findings of this study are available from the corresponding author upon reasonable request.
