# Supplementary material for: In Vitro Characterization of Biodegradable Polyurethane Foams With Facile Gelatin Modification for Traumatic Wound Hemostasis and Regeneration
Source: J Biomed Mater Res A. Author manuscript; Available in PMC 2026 Apr 7. (PMC13055500; doi:10.1002/jbm.a.37982)
Supplement: Supporting Information [file NIHMS2154596-supplement-Supporting_Information.docx]

**Supplemental Information for**

***In vitro* characterization of biodegradable polyurethane foams with facile gelatin modification for traumatic wound hemostasis and regeneration**

Natalie Marie Petryk^1^ and Mary Beth B. Monroe^1*^

^1^Biomedical and Chemical Engineering and BioInspired Syracuse: Institute for Material and Living Systems, Syracuse University, Syracuse, New York, 13244

*Corresponding author:

Dr. Mary Beth Browning Monroe

Department of Biomedical and Chemical Engineering

Bioinspired Institute for Material and Living Systems

Syracuse University

318 Bowne Hall

Syracuse, NY 13244

Tel: (315) 443-3323

E-mail: [mbmonroe@syr.edu](mailto:mbmonroe@syr.edu)

**
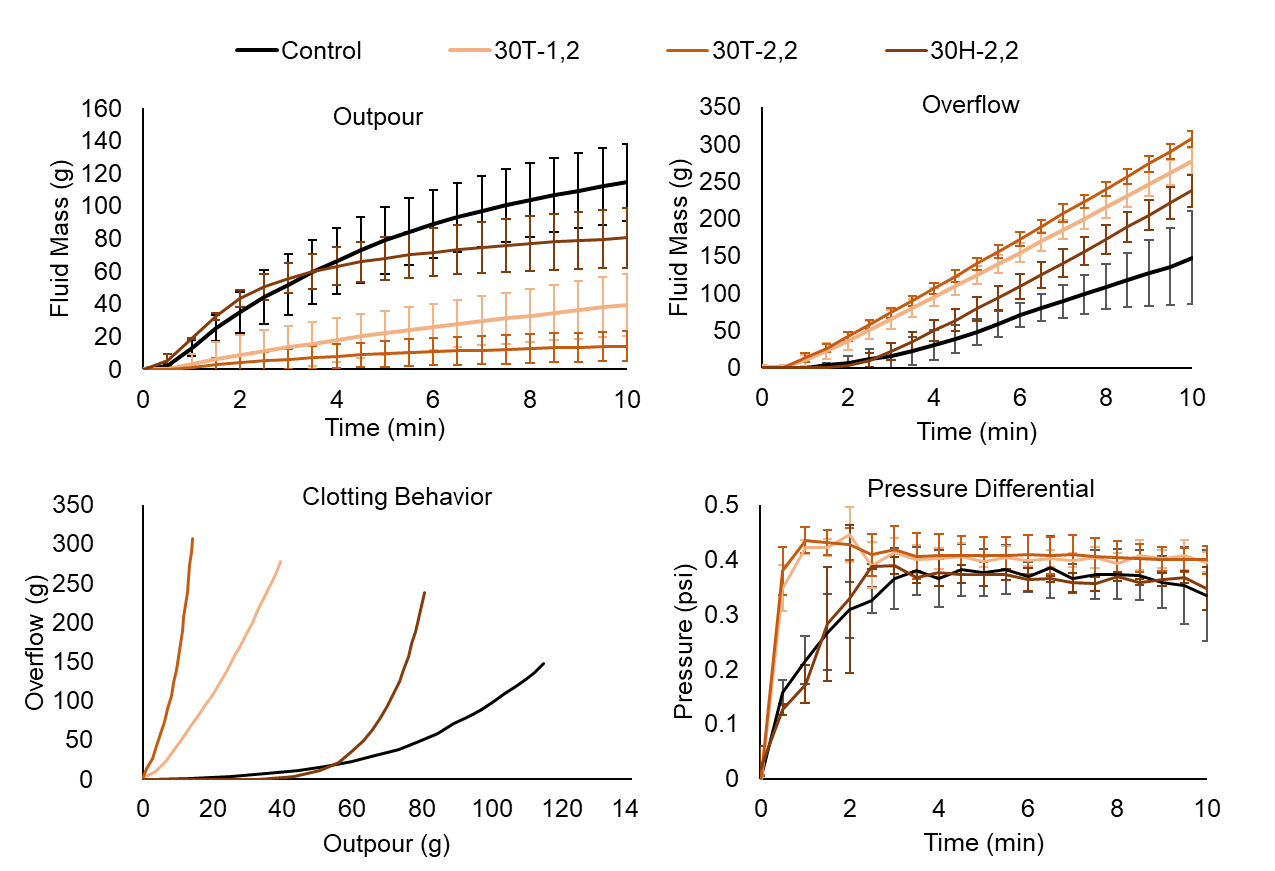
**

**Figure S1.** Dynamic flow summary demonstrating the clotting behavior of degradable PUr foams perfused with Na-citrated whole porcine blood for 10 minutes.


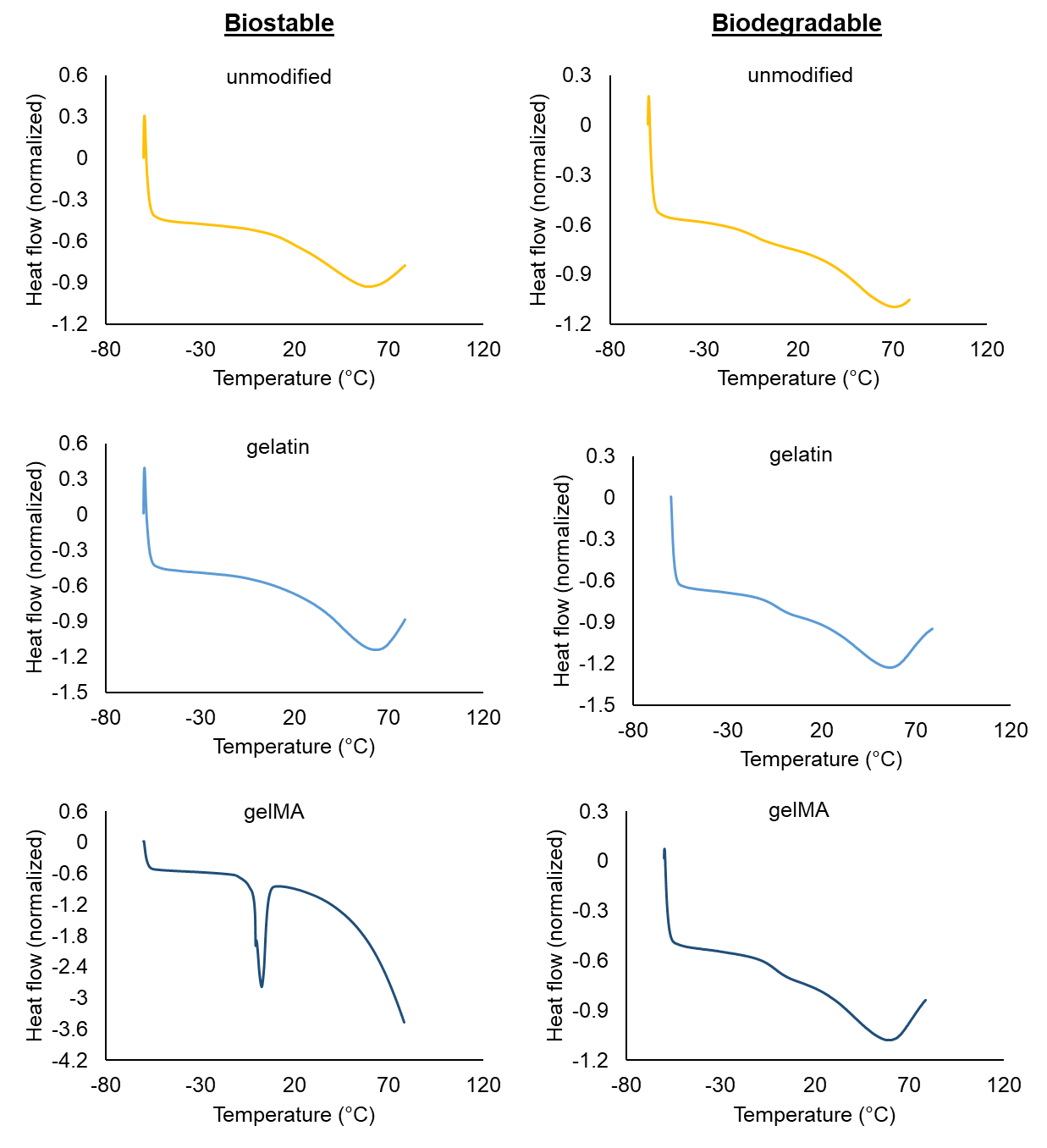


**Figure S2.** Representative differential scanning calorimetry (DSC) plots of wet (plasticized) samples.

**
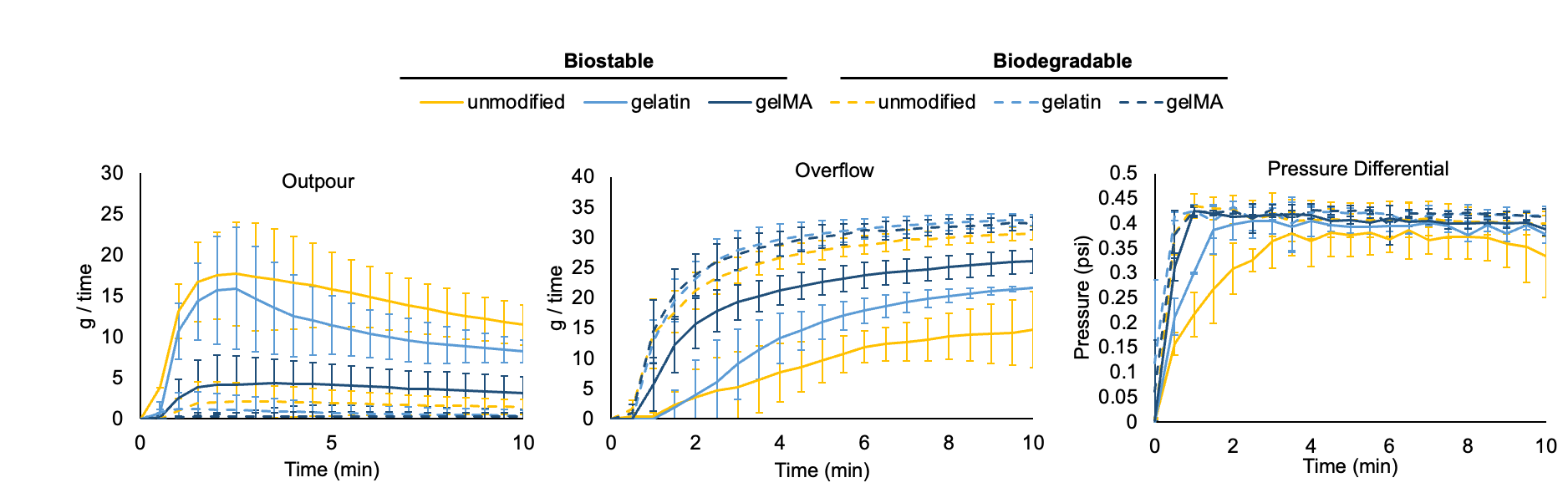
**

**Figure S3.** Flow rate changes over time of fluid collected in the outpour and overflow containers, and the pressure differential across each foam during the 10-minute perfusion runs with Na-citrated whole porcine blood.

**
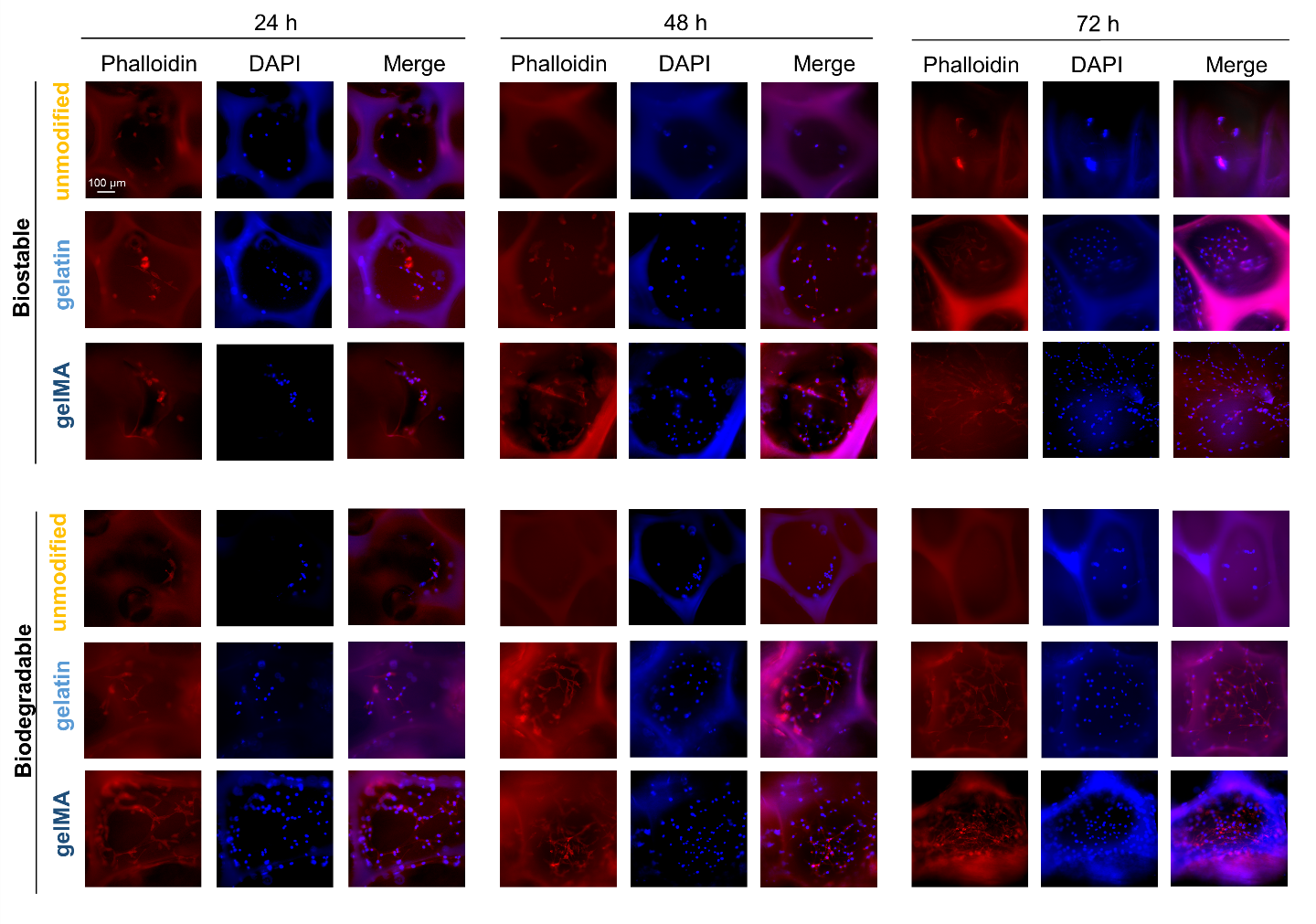
**

**Figure S4.** Separate red (phalloidin) and blue (DAPI) channels that make up the merged images of NIH/3T3 cells attached to the PUr foams.
